# Thyroid Carcinoma with Bone Metastases: A Prognostic Factor Study

**DOI:** 10.4137/cmo.s333

**Published:** 2008-02-09

**Authors:** Karl Wu, Shen-Mou Hou, Tien-Shang Huang, Rong-Sen Yang

**Affiliations:** 1 Department of Orthopedics, College of Medicine, National Taiwan University, National Taiwan University Hospital, Taipei, Taiwan; 2 Department of Internal Medicine, National Taiwan University, National Taiwan University Hospital, Taipei, Taiwan

**Keywords:** thyroid, carcinoma, bone, metastasis, prognosis

## Abstract

**Objective:**

Occult clinical presentations usually hinder the early detection and management of patients with bone metastases from thyroid carcinoma. We retrospectively investigated the clinical outcomes of such patients from 1993 to 2004 and analyzed the prognostic parameters.

**Design:**

The basic demographic data and manifestations of 44 patients who had thyroid carcinoma with bone metastases were reviewed. We studied the gender, age, locations of metastases, histological types, treatment methods, hypercalcemic episodes and results of treatments. We used Kaplan-Meier survival analysis and log-rank tests to access the statistical significance.

**Main outcome:**

The incidence of bone metastasis from thyroid carcinomas in this series was 5.0%. Twenty patients (45.4%) had follicular, 16 (36.3%) had papillary, 3 (6.8%) had anaplastic, 3 (6.8%) had medullary, and 2 (4.5%) had Hurthel cell carcinomas. Twelve patients had hypercalcemic episodes, ranging from 2.6 to 2.9 mmolL^−1^ (mean ± SD: 2.68 ± 0.15 mmolL^−1^). Survival time after bone metastases ranged from 2 months to 8 years (mean ± SD: 5.3 ± 1.3 years). The 5-year survival rate was 79.4% and the 10-year survival rate was 52.9%. Regarding the histological cancer type, patients with papillary and follicular cancers survived significantly longer than those with anaplastic and medullary cancers (p < 0.05). In addition, the patients presenting with hypercalcemia had the worst survival (p < 0.05).

**Conclusions:**

Thyroid carcinoma can present with bone metastases in its early stage. We found that both tumor type and hypercalcemia were significant prognostic factors for survival time.

## Introduction

Carcinoma of the thyroid gland is one of the possibly curable cancers.^15^ At the time of initial diagnosis, 1%–3% of patients with thyroid cancer may have distant metastases, whereas another 7%–23% will develop distant metastases during the disease course.^3^,^14^,^16^ Yet, occult clinical presentations usually delay the early diagnosis and management of these metastases. The distant metastases, especially those involving bone, increase mortality rate, compromise quality of life and shorten patient survival.^9^,^14^

The imaging techniques to detect bone metastatic lesions include plain radiography, computed tomography (CT), magnetic resonance imaging (MRI), isotope bone scans, positron emission tomography (PET),^4^ radioiodine uptake, and others. The management of primary thyroid cancer is multidisciplinary, including surgeons, endocrinologists, nuclear medicine physicians, and radiotherapists.^19^ The 5-year survival rates for patients with bone metastases ranged from 53% to 84% in previous reports.^1^,^2^,^16^

Although the estimated incidence of thyroid cancer has increased by 14.6% over the past 40 years, the estimated death rate has fallen by 21%, probably as a result of earlier diagnosis.^18^ Furthermore, trends toward smaller tumor size, less advanced clinical stage, longer disease-free survival, and lower mortality rates after 1993 was also noted in Taiwan.^6^ This finding emphasizes the importance of its early detection and proper treatment. In this study, we investigated 44 patients with thyroid cancers and bone metastases that were treated in our hospital from January 1993 to June 2004. The aim of this study was to investigate the prognostic factors of the patients with bone metastases due to thyroid cancer.

## Materials and Methods

From January 1993 to June 2004, there were 887 patients with thyroid carcinomas were treated at author’s hospital. Forty-four patients (5.0%) of the 887 patients had bone metastases. The medical records of the 44 patients were reviewed. We investigated the follow-up period, gender, age, clinical manifestations, locations of metastases, treatment methods, hypercalcemia episodes and results of the treatment.

Thyroid carcinomas in this series were histopathologically classified as follicular, papillary, Hurthel, medullary and anaplastic carcinoma. Bone metastatic lesions were detected by using plain skeletal radiograms, computed tomography (CT), magnetic resonance imaging (MRI), isotope bone scan, or radioiodine uptake either on routine follow up or as directed by the patients’ symptoms. There were wide variations of clinical presentations, 31 (70.4%) patients presenting skeletal pain or neurological symptoms caused by vertebral metastatic lesions, 4 (9.09%) presenting pathological fractures in the extremities, 6 (13.6%) asymptomatic and were found to have bone metastases incidentally on the radioisotope scan. Thyroid origin of metastatic disease was confirmed by pathological report from surgical specimen or presence of radioiodine uptake. For the patients who had received thyroidectomized, we have reviewed their pathology report.

The patients were treated according to the following guidelines. Radioactive iodine ^131^I was given when bone lesions were detected using 5mCi ^131^I diagnostic scans. These patients were treated with ^131^I radiotherapy to the maximum permissible dose based on quantitative dosimeter studies.^5^,^17^ Twelve patients in our study were treated with ^131^I for metstatic lesion, with a mean cumulative dose of 319 ± 105 mCi (range: 31–502 mCi). ^131^I therapies were repeated if the follow-up ^131^I scan showed residual or persistent metastatic bone lesions. External radiotherapy was also applied to treat lesions that were painful and could not be removed by surgery. Local bone surgery was indicated only for decompression, open reduction and internal fixation of pathologic fractures, and tumor resection or for diagnostic biopsy. All these patients underwent post-operation ^131^I treatment. In general, these patients were followed up regularly, with 5 mCi ^131^I diagnostic scans, plain chest radiogram, and plain radiograms of metastatic bone areas performed every 6 months or one year.

We used Kaplan-Meier method to analyze the survival curves, and used the log-rank test to assess the statistical differences among the different groups with respect to survival functions. Survival time was defined as time of follow up after diagnosis of bone metastasis. A p value less than 0.05 was considered to be statistically significant.

## Results

The demographic data of the patients are shown in Table 1. The age at the time of diagnosis ranged from 12 to 85 years (mean 62.1 years). Thirty-three patients (75%) were females and 11 (25%) were males. The primary histological classification included 20 (45.4%) follicular, 16 (36.3%) papillary, 3 (6.8%) anaplastic, 3 (6.8%) medullary, and 2 (4.5%) Hurthel cell carcinomas (Table 1).

Among the total 887 patients treated, 137 (15.4%) patients had follicular carcinoma, 598 (67%) papillary, 44 (5%) anaplastic, 25 (2.8%) medullary and 25 (2.8%) Hurthel cell carcinoma. The original bone metastatic rate was estimated to be 14.5% (20/137) for follicular carcinoma, 2.7% (16/598) for papillary carcinoma, 6.8% (3/44) for anaplastic carcinoma, 12% (3/25) for medullary carcinoma and 8% (2/25) for Hurthel cell carcinoma.

Twelve patients were noted to have hypercalcemia episodes, ranging from 2.6 to 2.9 mmolL^−1^ (mean 2.68 mmolL^−1^). Their mean survival time was 9.57 months (range 2–16.2 moths).

As the presenting symptoms, 25 (56.8%) patients had pain as the initial presenting symptom, 8 (18.1%) with a protruding mass, 6 (13.6%) had cord compression, 4 (9.1%) with pathological fracture, and 7 (16.0%) had bone metastases incidentally found on radioisotope scan. All of the metastases showed osteolytic lesions on radiograms.

Twenty-three patients (52.2%) presented with bone metastases before the thyroid cancers were diagnosed. Among them, 17 (74.0%) patients had pain as the initial presenting symptom, 3 (13%) patients with pathological fracture, and 3 (13%) patients with metastases incidentally found on radioisotope scan. Twenty five patients (56.8%) had multiple sites of bone metastases noted from the initial work up studies. Vertebrae 23(52.2%), femur 9(20.4%), skull 7(16.0%), pelvis 7(15.9%), and clavicle 6(13.6%) were the most common sites of metastases.

Of the 44 patients, surgical treatment was performed in 17 patients: nine patients (5 lumbar-spine lesions, 4 thoracic-spine lesions) with vertebral metastasis underwent decompression laminectomy; 8 patients with pathological fracture (5 femur, 4 tibia, 3 at humerus) received open reduction and internal fixation. Four complications occurred, including 3 postoperative infections and postoperative hemorrhage. All 17 patients had symptoms relieved after operation.

**Figure 1:**
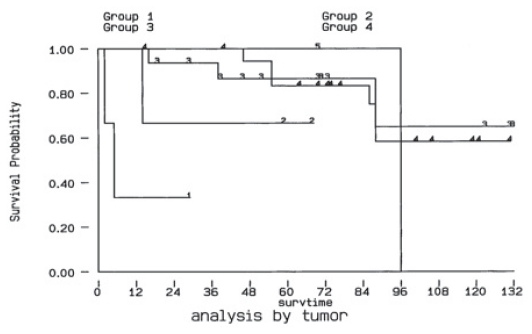
Survivor-ship curve as determined by Kaplan-Meier method, the 5-year survival rate was 79.4%, the 10-year survival rate was 52.9%.

**Figure 2:**
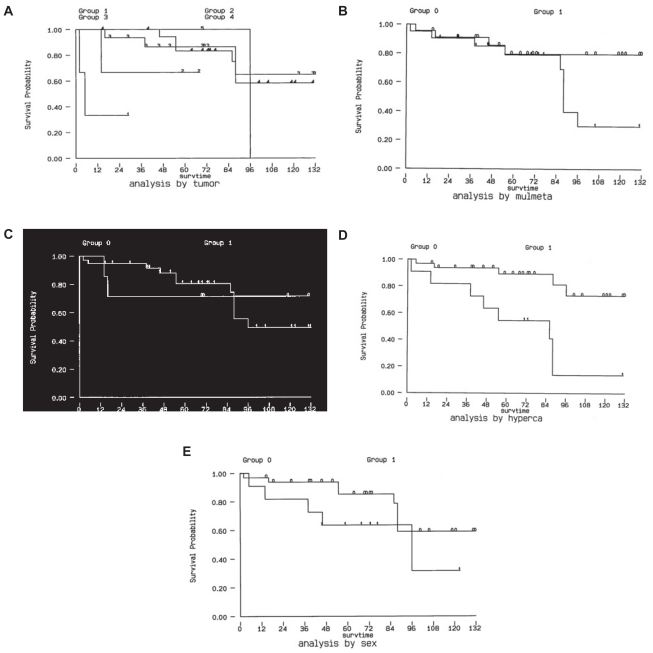
Kaplan – Meier plot of surviving after diagnosis of bone metastasis. The results showed that both tumor type and hypercalcemia were the significant prognostic factors (p < 0.05). (**A**) The 5-year survival rate was 64.2% for papillary cancer, 82.0% for follicular cancer, 0% for anaplastic cancer, 100% for Hurthel cell cancer and 61.1% for medullary cancer. (**B**) The 5-year survival rate for single bone metastasis was 81.2% and 80.4% for multiple bone metastases. (**C**) The 5-year survival rate 80.2% when treated with surgical method and 70.6% when treated without surgical method. (**D**) The 5-year survival rate for patients who have no hypercalcemia episode noted during the course of treatment was 84.0% and only 55.0% when more than one episode of hypercalcemia was noted. (p < 0.05) (**E**) The 5-year survival rate was 83.1% for female and 64.6% for male.

Using Kaplan-Meier method, the 5-year survival rate was 79.4%, the 10-year survival rate was 52.9% (Fig. 1). The 5-year survival rate was 64.2% for papillary cancer, 82% for follicular cancer, 0% for anaplastic cancer, 100% for Hurthel cell cancer and 61% for medullary cancer. With log rank analysis, we found that the patients with papillary cancer and follicular cancer significantly survive longer than those with the anaplastic type and medullary cancer (p < 0.05) (Fig. 2A). The patients presenting hypercalcemia had a worse survival (5-year survival rate 84% v.s. 55%) (p < 0.05) (Fig. 2D). The 5-year survival rate for single bone metastasis was 81.2% and 80.4% for multiple bone metastases, which didn’t show significant difference (Fig. 2B). The 5-year survival rate 70.6% when did not undergo any surgical treatment as compared with 80.2% for whom treated with surgical methods, which didn’t show significant difference, either (Fig. 2C). The 5-year survival rate also didn’t show any differnce between different genders. (female v.s. male, 83.1% v.s. 64.6%)(Fig. 2E).

On the other hand, gender, age, clinical manifestations, locations of metastases, and treatment methods did not show significant difference by log rank analysis (Fig. 2A~E). Although the patients with multiple bone metastasis seemed to have a worse survival, the difference is not significant.

The mean survival time was 9.6 months (range, 2.0–16.2 months). The survival time after bone metastases ranged for 2 months to 8 years (mean ± SD: 5.3 ± 1.3 years).

## Discussion

Bone metastases from aggressive thyroid carcinoma may cause serious complications. Unfortunately, the occult clinical presentations usually delay the early diagnosis and proper management. We conducted a retrospective analysis of prognostic factor in 44 patients with thyroid cancers and bone metastases during the period of January 1993 to June 2004. The results showed the significant prognostic values of hypercalcemia and bone tumor types.

Of the 887 patients with thyroid carcinomas treated at author’s hospital, 44 patients (5.0%) had bone metastases. The overall prevalence rate was similar to previous reports (3.8%–4.2%).^7^,^11^ Female patients (75%) were also more common than male (25%) as in previous reports (female, 82.7%; male 17.9%).^2^

The higher occurrence rate of bone metastases in follicular histotype (14.5%) than with papillary histotype (2.7%) has been observed in other series.^7^,^11^ The original bone metastatic rate in this series (14.5%) was similar in follicular carcinoma in the other series (15%–17%).^2^,^7^ However, bone metastases rate (2.7%) in papillary carcinoma was higher than that of Marcocci (0.6%).^7^ Differences in the referral pattern of patients may account for such discrepancy.

The distribution of osseous metastases in our series was similar to that observed by other authors with the spine and femur as the most common sites.^7^,^8^ Vertebrae 23(52.2%), femur 9(20.4%), skull 7(16.0%), pelvis 7(16.0%) and clavicle 6(13.6%) were the most common sites of metastases. Besides, multiple bone lesions occurred more frequently (56.8%) than single bone lesion, which was same to the report from Pittas and Fanchiag (53%; 79.5% respectively).^2^,^10^

As the initial presenting symptoms, 25(56.8%) patients had pain, 8(18.1%) patients with a protruding mass, six patients with cord compression, 4(9.1%) patients with pathological fracture, and 7(16.0%) patients had bone metastases incidentally found on radioisotope scan. This presenting symptoms and signs were similar to previous report.^2^

Patients with bone metastases of unknown origin are not rarely encountered, and the primary tumor is frequently found in the lung, breast, and prostate.^13^ It is worth noting that 23/44(52.2%) of our patients had the bone metastases as their initial presenting symptoms of thyroid cancer. The relative high incidence of bone metastasis in the patients with thyroid cancer makes it important to take it into consideration as a possible underlying lesion for those with skeletal metastatic diseases of unknown primary origin.

Surgical treatment was performed for 17/44 patients: nine patients with vertebral metastasis underwent decompression laminectomy; eight patients with pathological fractures underwent open reduction and internal fixation combined with palliative surgery. Although that surgical treatment was not a significant prognostic indicator in our study, it appears from these data that surgical treatment of metastases from thyroid cancer achieved good functional outcomes even in cases where neurological or mechanical complications had occurred. Other reports of surgical removal of respectable metastases also had favorable effects on the prognosis.^9^ Therefore, proper surgery to prevent or treat complications of metastatic bone disease appears to be a useful adjunct to improve morbidity in patients with skeletal disease.

The survival rate for patients with lung or bone metastases from thyroid gland was 53% at 5 year, 38% at 10 year, as described by Schlumberger et al.^16^ Ruegemer el al.^14^ found the 5 year survival rate to be 92% for age younger than 40 with a single organ metastatic lesion and a 5 year survival rate of 35% for those whose age were older than 40 with a single organ metastatic lesion form differentiated thyroid carcinoma. Using Kaplan-Meier method, the 5-year survival rate in our series was estimated to be 79.4%, and the 10-year survival rate was 52.9% (Fig. 1). A higher 5-year survival rate, and a higher 10-year survival rate in our series as compared with the report from Schlumberger et al.^16^ was noted (79.4 vs. 53%, 52.9 vs. 38%, respectively). The differences in the survival rate may be due to the different patient population and the different tumor subtype.

With log rank analysis, we found that tumor type and hypercalcemia were the prognostic factors (p < 0.05) (Fig. 2). Gender, age, clinical manifestations, locations of metastases, multiple bone metastasis and treatment methods did not show significant differences, which were compatible with the findings of previous reports.^10^ Poor life expectancy in cancer associated hypercalcemia was previously described by Stuart et al.^12^ However, thyroid cancer associated hypercalcemia has not been well described before. Our data indicated that hypercalcemia in patients with thyroid cancer and bone metastases signifies a grave prognosis. We could not find a correlation between hypercalcemia and thyroidectomized status. For the patients who had received thyroidectomized, we have reviewed their pathology report and none have shown to have coincidence parathyroid tissue within the specimen. It is still unclear about whether this association is due to death from hypercalcemic crises or to the fact that the development of hypercalcemia is simply a marker of tumor biological behavior, indicating more advanced disease.

There are several limitations to the present study. First, since these data collected where from a tertiary referral medical center, the prevalence rate and the presentations of the patients could be different than that from the general population. Second, the numbers of patients with anaplastic, medullary, and Hurthel cell carcinoma were limited, which made statistical evaluation difficult. Further study design with a larger number of subjects is necessary to confirm our results.

In conclusion, thyroid carcinoma can present with bone metastases in its early stage. Early detection and management should help disease control and improve patients’ quality of life. A high index of suspicion is needed to detect these early bone metastases at the initial visit, allowing prompt and thorough follow-up during clinical practice. Our data showed that hypercalcemia and original tumor cell type are the prognostic factors.

## Figures and Tables

**Table 1:** Demographic data of the patients with bone metastases form thyroid carcinoma.

	Follicular (N = 20)	Papillary (N = 16)	Anaplastic (N = 3)	Hurthel cell (N = 2)	Medullary (N = 3)	Total (N = 44)
Age	62.3	64.2	69.6	66.5	39.0	62.1
Female: Male	18:2	12:4	2:1	1:1	0:3	33:11
Pain	7	11	3	1	3	25 (56.8%)
Protruding mass	4	3	0	1	0	8 (18.1%)
Nerologic symptoms	1	2	0	0	0	6 (13.6%)
Fractures	3	1	0	0	0	4 (9.1%)
Hypercalcemia	4	6	1	0	1	12 (27.3%)
Multiple metastasis	13	7	2	2	1	25 (56.8%)

## References

[1] Chen MH, Chang CC, Huang TS (2003). Factors affecting long-term survival of Taiwanese patients with medullary thyroid carcinoma. J. Formos. Med. Assoc.

[2] Fanchiang JK, Lin JD, Huang MJ (1998). Papillary and follicular thyroid carcinomas with bone metastases: a series of 39 cases during a period of 18 years. Changgeng Yi Xue Za Zhi.

[3] Harness JK, Thompson NW, Sisson JC (1974). Proceedings: Differentiated thyroid carcinomas. Treatment of distant metastases. Arch. Surg..

[4] Iwata M, Kasagi K, Misaki T (2004). Comparison of whole-body 18F-FDG PET, 99mTc-MIBI SPET, and post-therapeutic 131I-Na scintigraphy in the detection of metastatic thyroid cancer. Eur. J. Nucl. Med. Mol. Imaging.

[5] Leeper RD (1973). The effect of 131 I therapy on survival of patients with metastatic papillary or follicular thyroid carcinoma. J. Clin. Endocrinol. Metab..

[6] Lin JD, Chao TC, Sun JH (2000). Trends in the clinical characteristics of patients with papillary thyroid carcinoma in Taiwan. Oncology.

[7] Marcocci C, Pacini F, Elisei R (1989). Clinical and biologic behavior of bone metastases from differentiated thyroid carcinoma. Surgery.

[8] McCormack KR (1966). Bone metastases from thyroid carcinoma. Cancer.

[9] Niederle B, Roka R, Schemper M (1986). Surgical treatment of distant metastases in differentiated thyroid cancer: indication and results. Surgery.

[10] Pittas AG, Adler M, Fazzari M (2000). Bone metastases from thyroid carcinoma: clinical characteristics and prognostic variables in one hundred forty-six patients. Thyroid.

[11] Proye CA, Dromer DH, Carnaille BM (1992). Is it still worthwhile to treat bone metastases from differentiated thyroid carcinoma with radioactive iodine?. World J. Surg..

[12] Ralston SH, Gallacher SJ, Patel U (1990). Cancer-associated hypercalcemia: morbidity and mortality. Clinical experience in 126 treated patients. Ann. Intern. Med..

[13] Rougraff BT (2003). Evaluation of the patient with carcinoma of unknown origin metastatic to bone. Clin. Orthop..

[14] Ruegemer JJ, Hay ID, Bergstralh EJ (1988). Distant metastases in differentiated thyroid carcinoma: a multivariate analysis of prognostic variables. J. Clin. Endocrinol. Metab..

[15] Schlumberger M, Baudin E, Travagli JP (1998). Papillary and follicular cancers of the thyroid. Presse Med..

[16] Schlumberger M, Tubiana M, De Vathaire F (1986). Long-term results of treatment of 283 patients with lung and bone metastases from differentiated thyroid carcinoma. J. Clin. Endocrinol. Metab..

[17] Van Nostrand D, Neutze J, Atkins F (1986). Side effects of “rational dose” iodine-131 therapy for metastatic well-differentiated thyroid carcinoma. J. Nucl. Med..

[18] Vini L, Harmer C (2002). Management of thyroid cancer. Lancet Oncol..

[19] Vini L, Harmer C, McCready VR (1996). Thyroid cancer: a review of treatment and follow-up. Ann. Nucl. Med..

